# Microstructural differences in the thalamus and thalamic radiations in the congenitally deaf

**DOI:** 10.1016/j.neuroimage.2014.05.077

**Published:** 2014-10-15

**Authors:** Rebecca C. Lyness, I. Alvarez, Martin I. Sereno, Mairéad MacSweeney

**Affiliations:** a Cognitive and Perceptual Brain Sciences, 26 Bedford Way, University College London, London WC1H 0AP, UK; b Institute of Child Health, University College London, London WC1N 1EH, UK; c Birkbeck, University of London, Malet Street, Bloomsbury, London WC1E 7HX, UK; d Deafness, Cognition & Language Research Centre, 49 Gordon Square, University College London, London WC1H 0PD, UK; e Institute of Cognitive Neuroscience, 17 Queen Square, University College London, London WC1H 3AR, UK

**Keywords:** Diffusion weighted MRI, Deafness, Thalamus, Neuroplasticity

## Abstract

There is evidence of both crossmodal and intermodal plasticity in the deaf brain. Here, we investigated whether sub-cortical plasticity, specifically of the thalamus, contributed to this reorganisation. We contrasted diffusion weighted magnetic resonance imaging data from 13 congenitally deaf and 13 hearing participants, all of whom had learnt British Sign Language after 10 years of age. Connectivity based segmentation of the thalamus revealed changes to mean and radial diffusivity in occipital and frontal regions, which may be linked to enhanced peripheral visual acuity, and differences in how visual attention is deployed in the deaf group. Using probabilistic tractography, tracts were traced between the thalamus and its cortical targets, and microstructural measurements were extracted from these tracts. Group differences were found in microstructural measurements of occipital, frontal, somatosensory, motor and parietal thalamo-cortical tracts. Our findings suggest that there is sub-cortical plasticity in the deaf brain, and that white matter alterations can be found throughout the deaf brain, rather than being restricted to, or focussed in the auditory cortex.

## Highlights

• Diffusion weighted MRI data was acquired from congenitally deaf and hearing groups.
• Mean and radial diffusivity is altered in the thalamus of congenitally deaf people.
• Diffusivity in white matter thalamo-cortical radiations is altered in deafness.

## Introduction

There is evidence of a number of different plastic processes in the deaf brain, which occur in response to, and to compensate for the atypical sensory environment. These include crossmodal (Fine et al., 2005, Finney et al., 2001, MacSweeney et al., 2004, Nishimura et al., 1999, Petitto et al., 2000), and intermodal plasticity (Bottari et al., 2011, Buckley et al., 2010, Codina et al., 2011), in addition to the dystrophic changes which occur in the auditory cortex (Emmorey et al., 2003, Li et al., 2012). The thalamus is an important structure for regulating both the flow of information into the cortex and between cortical areas. Whether this structure is altered in congenitally deaf humans has not yet been investigated.

Crossmodal plasticity is evident in the congenitally deaf brain. Activation in the secondary auditory cortices has been robustly demonstrated in fMRI studies in response to a wide range of visual stimuli, including sign language (MacSweeney et al., 2002, Petitto et al., 2000), biological motion (MacSweeney et al., 2004), as well as more simple visual stimuli such as dot motion (Finney et al., 2001). Controversy remains as to whether there is visual colonisation of Heschl’s gyrus, the typical site of primary auditory cortex. In deaf people, activation in response to visual stimuli has been reported in studies using spatial normalisation procedures (Finney et al., 2001), and in studies which do not contrast visual stimuli to a resting baseline (Karns et al., 2012, Scott et al., 2014). However, Cardin (2013) did not find activation in a cytoarchitectonically based definition of primary auditory cortex when visual stimuli were contrasted to a resting baseline in deaf participants.

Somatosensory processing has been shown to be enhanced (Levanen and Hamdorf, 2001), and reorganised into auditory cortex in deaf people (Auer et al., 2007, Karns et al., 2012, Levanen et al., 1998). The use of spatial normalisation to a common template for MRI data (Auer et al., 2007), and MEG data (Levanen et al., 1998) preclude confident anatomical localisation of this activation to primary auditory cortex. However, when anatomical definitions of the regions are used, there is strong evidence of somatosensory takeover of primary auditory cortex (Karns et al., 2012). Findings from the animal literature concur with this also (Allman et al., 2009, Meredith et al., 2012). Single unit recordings from the auditory cortex of early deafened ferrets (oto-toxic lesions) have demonstrated somatosensory afferents in auditory cortex (Meredith and Allman, 2012). Tracer injections to the auditory core of these deafened animals revealed the same auditory thalamo-cortical projection sources as the hearing ferrets, which the authors interpreted as indicating that rather than new or unmasked latent projections, reorganisation occurred at the level of the brainstem (Meredith and Allman, 2012).

In addition, there is evidence of intermodal plasticity in deafness. Deafness enhances detection of both static and motion targets in the visual periphery (Loke and Song, 1991, Neville and Lawson, 1987b). This behavioural advantage is thought to facilitate the orienting to targets in the absence of sound (Merabet and Pascual-Leone, 2010). These changes have been linked to increases in the area of neural rim within the optic nerve head, and thicker retinal nerve fibre layer in temporal (peripheral) retina (Codina et al., 2011), and changes in primary visual cortex (Lyness et al., 2013). Differences in visual event-related potentials (ERPs) have also been observed in early visual cortex in deaf groups, which in turn were correlated with improved performance in a visual target detection task (Bottari et al., 2011).

That the function of a brain region is tightly coupled with its extrinsic anatomical connections is a widely held assumption in neuroscience. It follows that the inputs to a region affect what information is available to a region, and where the outputs of a region terminate determines the influence that a region will have. Empirical tests of this hypothesis have supported this assumption (Passingham et al., 2002, Saygin et al., 2011), and indeed, anatomical connectivity data can be used to define functionally distinct regions (Behrens et al., 2003, Behrens et al., 2006, Johansen-Berg et al., 2004, Rushworth et al., 2006). Thus we argue that functional imaging studies concerning plasticity as a result of deafness should be considered in the context of changes to anatomical connectivity patterns. This complimentary approach may elucidate why certain patterns of reorganisation are seen in one brain region or modality, but not others.

Plastic change in the deaf brain may occur via a number of different mechanisms, none of which are mutually exclusive, and are likely have a different impact depending on the brain region (Bavelier and Neville, 2002). For example, visual activation in secondary auditory cortices may occur through synaptic reweighting of these regions, which typically act as a site for audiovisual integration (Calvert et al., 2000, Lee and Noppeney, 2011, McGettigan et al., 2012). Alternatively, the ‘brainstem theory of crossmodal reorganisation’ proposes that neither new nor latent projections are responsible for reorganisation, but instead, somatosensory inputs are able to takeover dormant auditory inputs found in the typically developing auditory brainstem at several nodes (Meredith and Allman, 2012). Subcortical connectivity changes have been suggested to contribute to crossmodal reorganisation as a result of congenital deafness, however, research into this possibility has as yet been limited to animal studies (see Proksch and Bavelier, 2002).

Here, we investigate how congenital deafness affects the thalamus, and thalamo-cortical projections. The thalamus has a critical role in regulating the flow of information into the cortex, as a substantial amount of information coming into the cortex does so through the thalamus (Sherman, 2007). In addition, and perhaps more importantly, the thalamus mediates cortico-thalamo-cortical connections, which make it ideally positioned functionally and anatomically to modulate a variety of different cognitive functions, which include emotion, motivation and multimodal perception (Jones, 2009, Sherman, 2007). Based on the overlapping nature of projections from different sensory modalities, the thalamus has additionally been suggested as a site of multimodal interplay (Cappe et al., 2009a, Cappe et al., 2009b). This has led to recent interest in the functional consequences of thalamic stroke (Carrera and Bogousslavsky, 2006), and the role of the thalamus in neurodevelopmental disorders such as autism spectrum disorder (Nair et al., 2013). Therefore, it is possible that looking at changes to the anatomy of the thalamus and thalamo-cortical tracts may illuminate the functional consequences of auditory deprivation.

Diffusion weighted magnetic resonance imaging (DW-MRI) is currently the only method for characterising neural tissue microstructure and reconstructing white matter tracts in vivo. Magnetic field gradients are used to sensitise the MRI signal acquisition to the displacement of water molecules due to Brownian motion. The application of diffusion gradients along multiple geometric directions allows the estimation of directional molecule displacement in the tissue sampled (Johansen-Berg and Rushworth, 2009). These data can be summarised by a diffusion tensor model, which describes the magnitude of the three principal axes of molecule displacement at each voxel sampled. Diffusion of water molecules is hindered by tissue properties, and in the case of white matter these include (but are not specific to) axonal ordering, axonal density and the degree of myelination (Johansen-Berg and Behrens, 2006). These underlying tissue properties can be approximated using tensor-derived microstructural metrics. These include fractional anisotropy (degree to which the first eigenvector dominates the second two), mean diffusivity (overall water diffusion in the specific voxel), and radial diffusivity (diffusion perpendicular to the principal eigenvector of the diffusion tensor).

Tractography with DW-MRI involves reconstructing continuous long range trajectories from voxel-wise estimates of the fibre orientation (Jones et al., 2013). From a seed region, streamlines can be traced in a probabilistic iterative fashion to determine the most likely path of the white matter tract of interest (Behrens et al., 2003). Tractography can be used to determine whether tracts exist between regions, and also to compare tracts in terms of their microstructural properties between groups (Johansen-Berg and Rushworth, 2009). Additionally, connectivity based segmentations of anatomical structures can be completed, in which structures are segmented on the basis of the highest probability of connection with different anatomical targets (Behrens et al., 2003). Behrens et al., first demonstrated this by generating a connectivity based segmentation of the thalamus, which closely resembled those derived from both animal anatomical tract tracing studies (Jones, 1985), and histological analyses (Morel et al., 1997).

DW-MRI data only detects the axis of diffusion (Johansen-Berg and Rushworth, 2009), and so we cannot differentiate between anatomical connections carrying information from the thalamus to its cortical targets (thalamo-cortical feedforward connections) from those carrying information from cortical targets to the thalamus (cortico-thalamic feedback connections). For simplicity, and to indicate that we have traced from thalamus to cortex, throughout this paper we refer to these tracts as thalamo-cortical connections with the understanding that they are likely to incorporate both feedforward and feedback connections.

To investigate the possible influence of congenital deafness on the anatomy of the thalamus, we first parcellated the thalamus based on connectivity profiles with its primary cortical targets. We contrasted the scalar microstructural measures of fractional anisotropy (FA), mean diffusivity (MD), and radial diffusivity (RD) in each parcellation between deaf and hearing groups. Second, to investigate the possibility of altered thalamo-cortical connectivity in congenital deafness, we reconstructed the tracts between the thalamus and its primary cortical targets, extracted microstructural measures from each of these tracts, and then contrasted these between deaf and hearing groups.

## Method

### Participants

Thirty right-handed participants were scanned. Fifteen were congenitally deaf and 15 were hearing. Deaf participants were either severely or profoundly deaf in both ears. All participants were screened to ensure that they had no previous neurological or psychiatric history, current health problems, and were not taking psychoactive medication. One male deaf participant was excluded due to excessive motion artefacts, and a further deaf and a hearing male were excluded due to poor image quality. One hearing female participant was found to have an arteriovenous malformation, and was excluded from further analysis. This left 13 hearing (10 female) and 13 deaf (7 female) participants. For the 13 deaf participants, 5 were deaf through maternal rubella, 3 reported genetics as their cause of deafness, and 5 had an unknown cause of deafness. As vascular lesions causing intellectual disability can also occur as a result of maternal rubella, all images were screened by one of the authors who is an experienced neuroanatomist (MIS). No other neuroanatomical anomalies were detected. Furthermore, all deaf participants were either in skilled employment or higher education at the time of testing. The groups (following exclusion) did not differ in terms of age (*t*(*24*) = − *0.11, p = 0.921*, hearing mean 38.7(sd = 8.1), deaf mean 39.08 (sd = 11.08)).

Here, we study deaf people who did not learn British Sign Language (BSL) until 10 years of age, as previous studies of the neural bases of visual motion processing have reported an interaction between the influence of deafness and native acquisition of sign language (Bavelier et al., 2001, Neville and Lawson, 1987a). All deaf participants were born to hearing parents. To control for the effect of having learnt a visual manual language, we recruited hearing participants who had also learnt BSL after the age of 10. The deaf group was younger than the hearing group when they began to learn (*t*(*24*) = *3.263*, *p = 0.003*, hearing mean 25.6 (sd = 7.63), deaf mean 17.29 (sd = 4.68)). Many of the hearing group used BSL in a professional context as interpreters, teachers of the deaf or researchers in the field. With regard to language use before exposure to BSL, of the 13 deaf participants, 11 reported that they could fluently converse with hearing people in everyday situations through the use of lip-reading. This suggests that for these deaf participants, spoken English was used as a robust and secure first language. The remaining 2 reported that they were unable to make use of speechreading in everyday situations, which indicates that they may have insecure first language development. We additionally completed the analyses excluding these participants, in order to test whether they were driving any observed effects. None of the participants were educated in BSL. Eleven deaf participants reported that they were educated via spoken language only, whereas 2 reported that their school made use of sign supported English (using manual signs to support spoken English).

The study was approved by UCL Ethics Committee and the participants provided informed consent.

### Imaging protocol

Data acquisition was carried out at the Birkbeck UCL Centre for Neuroimaging using a 1.5T Siemens Avanto MRI scanner (Erlangen, Germany). Diffusion weighted images were acquired using a diffusion weighted EPI sequence (TR = 7500 ms TE = 104 ms) with a 32 channel head coil. Whole brain volumes were acquired with 46 contiguous axial slices. Voxel size was 2.3 mm^3^. Diffusion-sensitizing encoding gradients were applied in 64 directions (b = 1000s/mm^2^) and 1 volume was acquired without diffusion weighting (b = 0 s/mm^2^).

Two diffusion weighted scans were acquired from the participants in all instances, apart from one female hearing participant who had her second scan aborted due to reporting shoulder pain.

An MPRAGE structural sequence with voxel size of 1 mm^3^, flip angle of 7°, T1 = 1000 ms, TR = 8.4 ms, TE = 3.57 ms and BW = 190 Hz/pix was acquired, also by using the 32 channel head coil.

### Image analysis

Cortical reconstruction was completed by using FreeSurfer 5.0.0 (http://surfer.nmr.mgh.harvard.edu/). Comprehensive details of these procedures are provided in previous publications (Dale et al., 1999, Fischl et al., 1999a, Fischl et al., 1999b, Fischl and Dale, 2000, Fischl et al., 2001, Fischl et al., 2002, Fischl et al., 2004, Han et al., 2006, Jovich et al., 2006, Segonne et al., 2004). Briefly, brightness and contrast normalisation is performed on the images, and then all non-brain tissues are removed with a hybrid watershed/surface deformation procedure (Segonne et al., 2004). Images then undergo Talairach transformation, subcortical white matter and deep grey matter structures are segmented (Fischl et al., 2004), the grey white matter boundary is tessellated, topology automatically corrected (Fischl et al., 2001, Segonne et al., 2007), and surface deformation is performed by using intensity gradients to optimally place the grey/white and grey/CSF borders where the greatest change in intensity signifies transition to the other tissue class (Dale et al., 1999).

### DW-MRI pre-processing

All processing and analysis of DW-MRI data were completed in FSL 5.0 (http://fsl.fmrib.ox.ac.uk/fsl/fslwiki/). Eddy current and movement correction were completed with the FMRIB Diffusion Toolbox (FDT). Following this, the two DW-MRI scans taken of each participant were averaged by taking the arithmetic mean of each voxel across scans. Each individual's structural T1 image was registered with their diffusion data using the FMRIB Linear Image Registration Tool (FLIRT). DTIFIT was then used to fit a diffusion tensor model and generate FA, MD and RD maps, and the BEDPOSTX toolbox was used subsequent to this to fit a ball-and-stick model to the data. The complexity of underlying tissue structure can be estimated, and this information incorporated in a Bayesian manner into a crossing fibres model to account for situations in which two fibre bundles cross within a voxel (Behrens et al., 2007). This algorithm runs Markov Chain Monte Carlo sampling to build up distributions of diffusion parameters at each voxel, enabling the modelling of crossing fibres within a voxel, and the number of crossing fibres present in each voxel (Behrens et al., 2007).

### Regions of interest

**Fig. 1:**
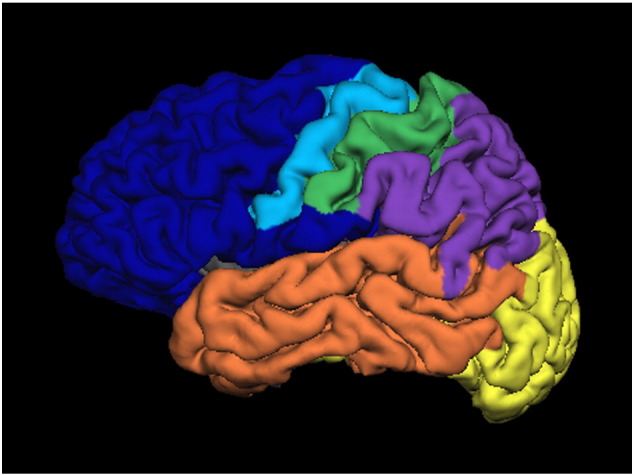
Cortical target masks are demonstrated in a representative participant. The cortex has been divided into frontal (dark blue), motor (light blue), somatosensory (green), parietal (purple), temporal (orange) and occipital (yellow) regions.

The FreeSurfer cortical and subcortical segmentation was used to generate regions of interest (ROI). Specifically, the thalamus label generated in either hemisphere was used for the seed mask. A total of 6 target masks were used, which included occipital, temporal, parietal and frontal lobes, in addition to somatosensory cortex and motor cortex, analogous to cortical targets for thalamic parcellation in Behrens et al. (2003). Labels generated from the FreeSurfer cortical reconstructions were merged to form these regions, as demonstrated in Fig. 1. Specific labels from the Destrieux atlas in FreeSurfer in each parcellation are detailed in Table 1. These masks were additionally registered to the diffusion data using FLIRT, and subsequently binarised in order to carry out the tractography procedures.

**Table 1:** Freesurfer labels from the Destrieux atlas which were merged from each hemisphere in order to form the cortical target.

Cortical target	Labels	
Occipital	• *h.S_oc_middle_and_Lunatus • *h.G_and_S_occipital_inf • *h.G_occipital_middle • *h.G_occipital_sup • *h. h.G_oc-temp_lat-fusifor • *h.Pole_occipital • *h.G_cuneus • *h.G_oc-temp_med-Lingual	• *h.S_calcarine • *h.S_collat_transv_post • *h.S_oc_middle_and_Lunatus • *h.S_oc_sup_and_transversal • *h.S_occipital_ant • *h.S_oc-temp_lat • *h.S_oc-temp_med_and_Lingual
Parietal	• *h.S_subparietal • *h.G_parietal_sup • *h.G_pariet_inf-Supramar • *h.G_precuneus • *h.S_parieto_occipital • *h.G_pariet_inf-Angular • *h.S_intrapariet_and_P_trans	
Temporal	• *h.G_temp_sup-G_T_transv • *h.G_temp_sup-Lateral • *h.G_temp_sup-Plan_polar • *h.G_temp_sup-Plan_tempo • *h.G_temporal_inf • *h.G_temporal_middle • *h.S_temporal_inf • *h.S_collat_transv_ant	• *h.S_temporal_sup • *h.S_temporal_transverse • *h.Pole_temporal • *h.S_interm_prim-Jensen • *h.Lat_Fis-post
Frontal	• *h.G_front_inf-Opercular • *h.G_front_inf-Orbital • *h.G_front_inf-Triangul • *h.G_front_middle • *h.G_and_S_frontomargin • *h.G_and_S_transv_frontopol • *h.G_rectus • *h.S_front_inf • *h.S_orbital_lateral • *h.S_orbital_med-olfact	• *h.S_orbital-H_Shaped • *h.Lat_Fis-ant-Horizont • *h.Lat_Fis-ant-Vertical • *h.S_front_middle • *h.G_front_sup • *h.G_orbital • *h.S_suborbital • *h.S_front_sup • *h.G_and_S_subcentral
Motor	• *h.G_precentral • *h.S_precentral-inf-part • *h.S_precentral-sup-part	
Somatosensory	• *h.S_central • *h.S_postcentral • *h.G_postcentral • *h.G_and_S_paracentral	

### Connectivity based segmentation of thalamus

The probtrackx software in FDT was used to generate probabilistic tracts from the seed ROI (thalamus) to the cortical target masks (occipital/parietal/temporal/motor zone/somatosensory/frontal). For every seed and target pair, 5000 streamlines were initiated, and a curvature threshold of 0.2 was set in order to prevent the generation of anatomically unlikely tracts. Step size was set to 0.5 mm, and the number of steps to 2000. To reduce the complexity (and resulting ambiguity) of the tractography, and as the thalamus is predominantly unilaterally organised, only ipsilateral thalamo-cortical connections were considered. An exclusion mask along the midline of the contralateral hemisphere was generated to prevent the crossing of tracts into this region.

Following this, segmentation was performed with a ‘winner takes all’ approach, whereby each voxel in the thalamus is classified based upon the cortical target with which it has the highest probability of being connected to. The parcellations generated from this were thresholded so that all tracts which did not have at least 3000 of the 5000 streamlines (60%) reaching the target where discarded, in order to remove all connections with a low associated probability. The resulting images were then used as ROIs to extract FA, MD and RD values.

### Thalamo-cortical tracts

In addition to the thalamic parcellations, we examined tracts between the thalamus and individual cortical targets. Grey matter is more isotropic than white matter, and as such, the signal to noise ratio is lower, making diffusion indices in regions such as the thalamus relatively insensitive in comparison to those measured in white matter. To keep the analysis of tracts independent from the analysis of the thalamic parcellations, we used the entire thalamus as the seed region (as opposed to the parcellation derived from the connectivity based segmentation). The same cortical target masks were used as before. Again, 5000 streamlines were initiated, a curvature threshold was set to 0.2, step size was constrained to 0.5 mm and number of steps to 2000. To ensure anatomical specificity of the tracts, we completed a ‘winner takes all’ segmentation of cortical white matter voxels, in which when a voxel appeared in more than one thalamo-cortical tract, it was removed from all thalamo-cortical tracts, apart from the tract with the greatest probability of connection (highest number of streamlines). The output of the tractography was thresholded at 60% to reduce the contribution to the microstructural analysis of voxels with low connection probability.

## Results

### Connectivity based segmentation of thalamus

**Fig. 2:**
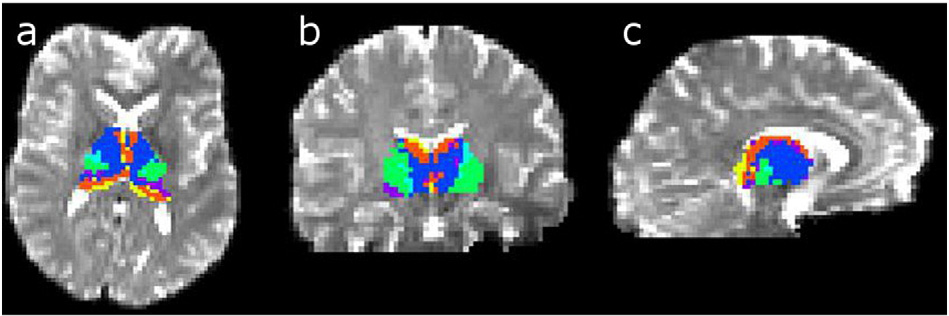
The connectivity based thalamic parcellation is demonstrated in; a) axial, b) coronal and c) sagittal views. The thalamus has been divided into frontal (dark blue), motor (light blue), somatosensory (green), parietal (purple), temporal (orange) and occipital (yellow) regions.

We first completed a connectivity based segmentation of the thalamus, using 6 cortical targets including occipital, parietal, temporal and frontal cortex, the motor zone and primary somatosensory area. An example of the thalamic parcellation is provided in Fig. 2. The thalamic parcellations generated here are comparable to those generated by other researchers using this method (Behrens et al., 2003).

To determine whether microstructural measures recorded from the same thalamic parcellation in either hemisphere were independent, and so should be treated as such in statistical analyses, we first correlated microstructural measurements from each parcellation measured in the right and left hemisphere. Table 2 shows the results of this analysis, which demonstrates that MD and RD measures are highly correlated. FA measures are correlated in the frontal parcellation, and there was also a trend towards correlation in the somatosensory parcellation. As such, we accounted for the non-independence of the hemispheres in the analyses.

**Table 2:** Correlation coefficient (R^2^) and p values are displayed for the correlation of microstructural measurements from parcellations in either hemisphere.

	Frontal		Motor		Somatosensory		Temporal		Parietal		Occipital	
	R^2^	p	R^2^	p	R^2^	p	R^2^	p	R^2^	p	R^2^	p
FA	0.4814	0.0128	0.1744	0.3942	0.3615	0.0696	0.1866	0.3614	0.1737	0.369	0.3187	0.1125
MD	0.8714	< 0.001	0.8829	< 0.001	0.9004	< 0.001	0.4067	0.0392	0.8589	< 0.001	0.5307	0.1125
RD	0.8775	< 0.001	0.8636	< 0.001	0.8369	< 0.001	0.4073	< 0.039	0.8526	< 0.001	0.5101	0.0078

For FA, MD and RD data, we used a repeated measures ANOVA with a between-subjects factor of group (deaf/hearing), 6 within-subjects factors of thalamic parcellation (occipital/temporal/parietal/motor/somatosensory/frontal), and modelled participants as random effects in order to account for correlated random errors between the hemispheres for each participant. For FA, there were main effects of group (*F*(*1,300*) = *4.71*, *p = 0.031*), parcellation (*F*(*5,300*) = *105.65*, *p < 0.001*), but no interaction between group and parcellation (*F*(*5,300*) = 1.59, *p* = *0.162*). For MD, there were main effects of group (*F*(*1,300*) = *13.61*, *p < 0.001*), parcellation (*F*(*5,300*) = *81.68*, *p < 0.001*), and an interaction between group and parcellation (*F*(*5,300*) = *5.41*, *p < 0.001*). Analysis of the RD measurements revealed that there were main effects of group (*F*(*1,300*) = *12.05*, *p = 0.001*), parcellation (*F*(*5,300*) = *92.08*, *p < 0.001*), and an interaction between group and parcellation (*F*(*5,300*) = *5.95*, *p < 0.001*). Thus microstructural measurements in thalamic parcellations differed between groups.

We further investigated these findings with post-hoc t-tests, the results of which are displayed in Table 3. The p values presented have had a false discovery rate correction (FDR) applied to control for multiple comparisons. This demonstrates that results were driven by the deaf group having increased MD and RD in both frontal and occipital thalamic parcellations. Table 4 shows mean values and standard deviations for microstructural measures for the groups in each thalamic parcellation.

**Table 3:** Microstructural measurements for each thalamic parcellation. T statistics and p values (with a FDR correction applied, α = 0.05) are provided, the degrees of freedom is 50 in all instances.

	Frontal		Motor		Somatosensory		Temporal		Parietal		Occipital	
	t	p	t	p	t	p	t	p	p	p	t	p
FA	1.4432	0.3791	− 1.7911	0.2380	− 1.8654	0.2380	− 1.3974	0.3791	− 0.8806	0.4985	0.1803	0.8577
MD	− 7.8439	**< 0.001**	0.6783	0.5647	0.8713	0.4985	− 0.5734	0.6024	− 0.9473	0.4985	− 3.5274	**0.0055**
RD	− 8.1209	**< 0.001**	1.0848	0.4985	1.1505	0.4985	− 0.6764	0.5647	− 1.0010	0.4985	− 3.4298	**0.0055**

**Table 4:** Mean (standard deviation) for hearing and deaf groups in microstructural measurements in thalamic parcellations.

	Frontal		Motor zone		Somatosensory		Temporal		Parietal		Occipital	
	Hearing	Deaf	Hearing	Deaf	Hearing	Deaf	Hearing	Deaf	Hearing	Deaf	H	D
FA	0.3458 (0.0202)	0.3371 (0.0252)	0.3954 (0.0666)	0.4251 (0.0521)	0.4135 (0.0479)	0.4338 (0.0278)	0.2966 (0.0249)	0.3093 (0.0393)	0.3468 (0.0283)	0.3556 (0.0420)	0.2767 (0.0501)	0.2744 (0.0379)
MD	0.0009 (0.0001)	0.0011 (0.0001)	0.0008 (0.0002)	0.0008 (0.0001)	0.0008 (0.0001)	0.0007 (0.000)	0.0012 (0.0002)	0.0012 (0.0002)	0.0008 (0.0001)	0.0009 (0.0002)	0.0011 (0.0002)	0.0013 (0.0002)
RD	0.0007 (0.0001)	0.0009 (0.0001)	0.0006 (0.0002)	0.0006 (0.0001)	0.0006 (0.0001)	0.0006 (0.000)	0.0010 (0.0002)	0.0010 (0.0002)	0.0007 (0.0001)	0.0007 (0.0002)	0.0010 (0.0002)	0.0012 (0.0002)

To discern whether results were influenced by two of the deaf participants potentially having insecure first language development, we repeated the analyses excluding these two participants. For FA, there were main effects of group (*F*(*1,276*) = *5.99*, *p = 0.015*), parcellation (*F*(*5,276*) = *101.05*, *p < 0.001*), and a trend towards a significant interaction between group and parcellation (*F*(*5,276*) = *2.07*, *p = 0.069*). For MD, there were main effects of group (*F*(*1,276*) = *11.8*, *p = 0.001*), parcellation (*F*(*5,276*) = *76.81*, *p < 0.001*), and an interaction between group and parcellation (*F*(*5,276*) = *5.98*, *p < 0.001*). For RD, there were main effects of group (*F*(*1,276*) = *10.76*, *p = 0.001*), parcellation (*F*(*5,276*) = *87.02*, *p < 0.001*), and an interaction between group and parcellation (*F*(*5,276*) = *6.64*, *p < 0.001*). Again, we followed up these results with post-hoc t-tests (Table 5), which revealed elevated MD and RD values in the deaf group in both frontal and occipital thalamic parcellations. This replicates the group results when these participants were included.

**Table 5:** Microstructural measurements for each thalamic parcellation when participants from the deaf group with insecure first language acquisition are excluded. T statistics and p values (with a FDR correction applied, α = 0.05) are provided, the degrees of freedom is 46 in all instances.

	Frontal		Motor zone		Somatosensory		Temporal		Parietal		Occipital	
	t	p	t	p	t	p	t	p	t	p	t	p
FA	1.1016	0.3827	− 2.2856	0.0970	− 1.8629	0.2066	− 1.2477	0.3827	− 0.8886	0.4546	0.2255	0.8226
MD	− 7.8008	**< 0.001**	1.329	0.3827	0.9257	0.4546	− 0.5932	0.5887	− 1.1617	0.3827	− 3.3680	**0.0078**
RD	− 8.0257	**< 0.001**	1.7213	0.2364	1.1487	0.3827	− 0.7257	0.5307	− 1.232	0.3827	− 3.3283	**0.0078**

### Thalamo-cortical tracts

**Fig. 3:**
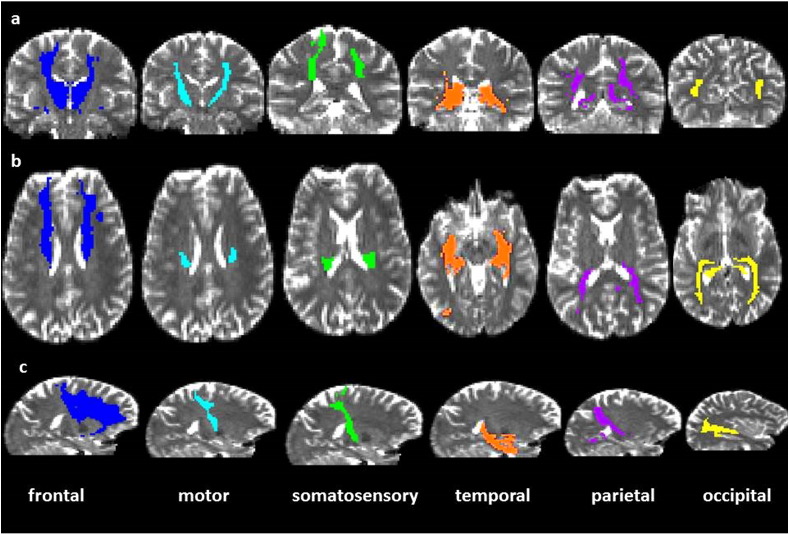
Each of the thalamo-cortical tracts is demonstrated in axial, coronal and sagittal slices; a) frontal, b) motor, c) somatosensory, d) temporal, e) parietal and f) occipital. Colour schemes are as in Fig. 1, Fig. 2.

As a second analysis, we calculated microstructural measures in the tracts between the thalamus and each of the cortical targets. Fig. 3 demonstrates these reconstructed tracts in a representative participant. Table 6 demonstrates that in the majority of tracts, diffusion measures for either hemisphere were highly correlated, and as such, we used a repeated measures ANOVA with between-subjects effects of group (deaf/hearing) and within-subjects effects of thalamo-cortical tract (occipital/temporal/parietal/motor/somatosensory/frontal), and to account for correlated random errors between each participants' hemispheres, modelled participants as random effects.

**Table 6:** Correlation coefficient (R^2^) and p values for the correlation between microstructural measurements in left and right hemisphere in all cortico-thalamic tracts.

	Frontal		Motor		Somatosensory		Temporal		Parietal		Occipital	
	R^2^	p	R^2^	p	R^2^	p	R^2^	p	R^2^	p	R^2^	p
FA	0.824	**< 0.001**	0.933	**< 0.001**	0.891	**< 0.001**	0.655	**< 0.001**	0.818	**< 0.001**	0.867	**< 0.001**
MD	0.751	**< 0.001**	0.776	**< 0.001**	0.675	**< 0.001**	0.623	**< 0.001**	0.695	**< 0.001**	0.397	**< 0.001**
RD	0.752	**< 0.001**	0.826	**< 0.001**	0.749	**< 0.001**	0.644	**< 0.001**	0.673	**< 0.001**	0.394	**0.046**

For FA, there were main effects of group (*F*(*1, 300*) = *61.19*, *p < 0.001*), tract (*F*(*5, 300*) = *22.53, p < 0.001*), and an interaction between group and tract (*F*(*5,300*) = *3.68*, *p = 0.003*). Analysis of the MD data revealed no main effect of group (*F*(*1,300*) = *1.24*, *p = 0.297*), but a main effect of tract (*F*(*5,300*) = *61.338*), and no interaction between tract and group (*F*(*5,300*) = *2.16*, *p = 0.059*). Finally, for the RD measures there were main effects of group (*F*(*1,300*) = *7.77*, *p = 0.006*), tract (*F*(*5, 300*) = *54.72*, *p < 0.001*) and an interaction between group and tract (*F*(*5,300*) = *2.35*, *p = 0.041*).

**Fig. 4:**
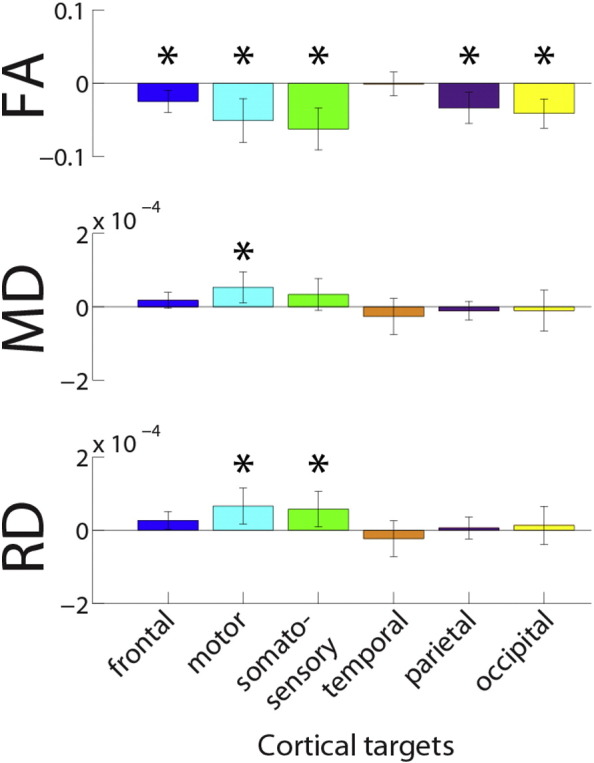
For microstructural measures in each of the thalamo-cortical tracts, the difference of the deaf group to the hearing group is displayed. Error bars denote confidence interval of the *t*-test statistic. Colour scheme is the same as Fig. 1, Fig. 2, Fig. 3.

Following this, we performed post-hoc t-tests to determine the source of the differences between groups; these results are presented in Table 7, and the mean and standard deviation of these tracts for each of the groups are presented in Table 8. Again, the p values presented have had a false discovery rate correction (FDR) applied to control for multiple comparisons. FA is reduced in the frontal thalamo-cortical tract in the deaf group. The motor thalamo-cortical tract is profoundly affected by deafness, with the deaf group demonstrating lower FA, increased MD and increased RD in this tract. The somatosensory thalamo-cortical tract is similarly affected, with decreased FA and increased RD in the deaf group. In both the parietal and occipital thalamo-cortical tracts, FA is reduced in the deaf group. These results are summarised in Fig. 4.

**Table 7:** T statistics and p values are shown for post hoc t tests on thalamo-cortical tracts. A FDR correction has been applied (α = 0.05), and the degrees of freedom is 50 in all instances.

	Frontal		Motor Zone		Somatosensory		Temporal		Parietal		Occipital	
	t	p	t	p	t	p	t	p	t	p	t	p
FA	3.3446	**0.0071**	3.4278	**0.0071**	4.4131	**0.0010**	0.1368	0.8918	3.1912	**0.0088**	4.1722	**0.0011**
MD	− 1.5819	0.2073	− 2.4871	**0.0418**	− 1.5533	0.2073	1.0803	0.4278	0.8570	0.5086	0.3689	0.7558
RD	− 2.2424	0.0588	− 2.6846	**0.0295**	− 2.3787	**0.0478**	0.9410	0.4863	− 0.443	0.7420	− 0.5225	0.7244

**Table 8:** Mean and standard deviations are presented for each of the microstructural measurements in each tract for hearing and deaf groups.

	Frontal		Motor zone		Somatosensory		Temporal		Parietal		Occipital	
	Hearing	Deaf	Hearing	Deaf	Hearing	Deaf	Hearing	Deaf	Hearing	Deaf	Hearing	Deaf
FA	0.3593 (0.0326)	0.3345 (0.0193)	0.3747 (0.0731)	0.3237 (0.0200)	0.4014 (0.0672)	0.3390 (0.0262)	0.3007 (0.0294)	0.2996 (0.0286)	0.3890 (0.0495)	0.3554 (0.0211)	0.3820 (0.0462)	0.3408 (0.0199)
MD	0.0008 (0.00004)	0.0009 (0.00004)	0.0008 (0.0001)	0.0009 (0.00004)	0.0008 (0.0001)	0.0009 (0.00005)	0.0010 (0.00008)	0.0010 (0.00009)	0.0008 (0.00006)	0.0008 (0.00003)	0.0010 (0.0001)	0.0010 (0.00009)
RD	0.0007 (0.00004)	0.0007 (0.00004)	0.0007 (0.0001)	0.0007 (0.00004)	0.0006 (0.0001)	0.0007 (0.00005)	0.0008 (0.00008)	0.0008 (0.00009)	0.0007 (0.00007)	0.0007 (0.00003)	0.0008 (0.0001)	0.0008 (0.00009)

Again, we completed the analysis excluding the two participants with insecure first language acquisition, and found for the FA values main effects of group (*F*(*1,276) = 53.07*, *p < 0.001*), tract (*F*(*5,276*) = *20.71*, *p < 0.001*), and an interaction between tract and group (*F*(*5,276*) = *2.52*, *p = 0.03*). For the MD values, there was no main effect of group (*F*(*1,276*) = *2.6*, *p = 0.108*), but a main effect of tract (*F*(*5,276*) = *55.5*, *p < 0.001*). There was no interaction between group and tract (*F*(*5,276*) = *1.53*, *p = 0.18*). For the RD values, there were main effects of group (*F*(*1,276*) = *9.39*, *p = 0.002*), tract (*F*(*5,276*) = *49.99*, *p < 0.001*), but no interaction between group and tract (*F*(*5,276*) = *1.55*, *p = 0.175*).

Post-hoc t-tests which are presented in Table 9 demonstrate that the frontal thalamo-cortical tract has decreased FA, and increased MD and RD in the deaf group. The motor thalamo-cortical tract has reduced FA, and increased MD and RD in the deaf group. FA is also decreased in the deaf group in the somatosensory, parietal and occipital thalamo-cortical tracts. The findings were comparable to when the entire group was analysed.

**Table 9:** T statistics and p values for microstructural measurements in each of the thalamo-cortical tracts, once the 2 participants who may not have secure first language development have been excluded. A FDR correction has been applied (α = 0.05), and degrees of freedom is 46 in all instances.

	Frontal		Motor		Somatosensory		Temporal		Parietal		Occipital	
	t	p	t	p	t	p	t	p	t	p	t	p
FA	3.4282	**0.0077**	2.9832	**0.0155**	3.8106	**0.0037**	0.4246	0.7219	3.1046	**0.0147**	3.9812	**0.0037**
MD	− 2.3777	**0.0484**	− 2.3306	**0.0484**	− 1.4557	0.2492	0.5413	0.7219	0.4909	0.7219	0.2065	0.8373
RD	− 2.9366	**0.0155**	− 2.4683	**0.0446**	− 2.1122	0.0722	0.4127	0.7219	− 0.6403	0.7219	− 0.6138	0.7219

## Discussion

From previous studies there is evidence of plasticity throughout the deaf brain. This includes crossmodal plasticity, in which visual and somatosensory stimuli come to be processed in auditory cortex (Auer et al., 2007, Fine et al., 2005, Finney et al., 2001, Karns et al., 2012, Levanen et al., 1998, MacSweeney et al., 2004, Nishimura et al., 1999), and intermodal plasticity (Bottari et al., 2011, Buckley et al., 2010, Codina et al., 2011), whereby the visual system is altered to compensate for hearing loss. In addition to this, there are dystrophic changes in auditory cortex (Kim et al., 2009, Li et al., 2012). In this study, we show that following connectivity based segmentation of the thalamus, the microstructural measurements of mean diffusivity (MD), and radial diffusivity (RD), were increased in the deaf group in the frontal and occipital thalamic parcellations. The thalamus supports many functions, including relaying information to the cortex, modulating the communication between different cortical areas through its extensive two-way connections with cortical regions, and is suggested to be a site of multimodal interplay. Thus our findings of differences in diffusion measurements between deaf and hearing participants in thalamic parcellations suggest that congenital deafness affects communication throughout the brain. Microstructural measurements were affected in the thalamo-cortical tracts to frontal, somatosensory, motor, parietal and occipital cortical targets. Changes to the microstructural measurements in the reconstructed tracts between the thalamus and its cortical targets additionally suggest differences in the flow of information throughout the cortex.

The mapping between DW-MRI diffusion tensor data and brain microstructure is a complex non-linear problem, which requires certain assumptions and provides no unique solution (Jones et al., 2013). Voxel-wise diffusion measures generated during the course of fitting the tensor model do not correspond directly to the anatomical features of potential interest, such as membrane integrity, axon diameter, axon count, myelin thickness and packing density of cells (Johansen-Berg and Rushworth, 2009). Therefore the biological significance of these metrics can be unclear. Nevertheless, we can interpret differences between groups in these microstructural measurements in light of findings from both the anatomical literature in animals and functional imaging studies with deaf participants. This enables us to draw tentative inferences about what underlying differences in grey and white matter tissue may be responsible for the differences in diffusion that we have found.

Recently, the increased ability of deaf people to be able to detect motion and static targets in the visual periphery has been linked to visual plasticity. Increased neuroretinal rim area (which is thought to be linked to increased retinal ganglion cell number) has been demonstrated in deaf participants, as well as thicker retinal nerve fibre layer in peripapillary regions which correspond to the temporal retina (Codina et al., 2011). These changes are linked to changes in visual field size as measured by Goldmann Perimetry (Codina et al., 2011). The optic nerve projects to the lateral geniculate nucleus of the thalamus, which projects to visual cortex. Previous studies have shown alterations in FA in the forceps major and splenium of the corpus calloseum at the site of inter-hemispheric connections between visual cortices (Kim et al., 2009, Li et al., 2012), suggesting that deafness affects connectivity in the visual system. Here, in the occipital thalamic parcellation, both MD and RD were increased in the deaf group. An increase in MD corresponds to an increase overall in the amount of diffusion which occurs in each voxel, and the concomitant increase in RD indicates that this is a result of increased diffusion in the axis parallel to the principal direction of diffusion. The optic thalamo-cortical tract additionally exhibited reduced FA. These changes may suggest increased tissue complexity in these regions. It is possible that these unexpected changes are linked to the enhanced peripheral acuity and visual field size reported in deaf people.

The fronto-parietal attention network is implicated in the top down modulatory signals to both the thalamus and early sensory areas (Gilbert and Sigman, 2007). Information in each of these regions then competes for representation in working memory in pre-frontal cortex, which in turn is implicated in attentional selection signals (Buschman and Miller, 2007, Miller and Buschman, 2013). A role for the lateral intraparietal area in generating a spatial priority map through behavioural prioritising of stimuli in a modality independent manner has also been posited (Bisley and Goldberg, 2010). Thus the increased MD and RD in the frontal thalamic parcellation and decreased FA in the frontal and parietal thalamo-cortical tracts in the deaf group may reflect the instantiation of altered attentional control and multimodal perception in the deaf brain.

The ‘brainstem theory of crossmodal reorganisation’ posits that in deafness, somatosensory afferents commandeer inert auditory afferents in auditory brainstem (Meredith and Allman, 2012). This results in crossmodal reorganisation, without the generation of new projections. We find no evidence of changes to somatosensory or auditory thalamus, which is consistent with this idea. Whilst it is problematic to interpret a null result, findings of significant alterations to frontal and occipital thalamus indicate that the methods can be sensitive to microstructural differences in the populations studied. The somatosensory thalamo-cortical tract has decreased FA and increased RD in the deaf group. These findings may be the anatomical correlate of there being an enhanced and more spatially distributed somatosensory representation in the deaf brain.

Somewhat counter-intuitively, we do not find differences between the deaf and hearing groups in the temporal thalamic parcellation, or thalamo-cortical tract. Decreased FA has been reported in deaf people in superior temporal regions, as well as white matter volume reductions in superior temporal gyrus, and temporal sub-gyral areas (Kim et al., 2009). Li et al. (2012) followed up by contrasting congenitally deaf participants and acquired deaf participants to hearing controls. In auditory cortex, they report reduced FA values bilaterally in superior temporal cortex (Li et al., 2012). These findings are correlated with the age of onset of deafness, as opposed to the duration of deafness, which the authors interpret as being indicative of an early sensitive period for typical development of auditory cortex (Li et al., 2012). There are reasons why our findings might diverge. First, the regions of interest between these studies are different, and so the results are not directly comparable: it remains a possibility that were we to study these regions of interest in auditory cortex there would be differences between the groups. On the other hand, in both these studies, deafness and language differences between the groups are conflated. No information is provided on language background by Kim et al. (2009), whereas in Li et al. (2012), all deaf participants used a sign language as their primary language whilst none of the hearing control participants had any knowledge of sign language. Bilingualism and language deprivation have both been shown to affect neuroanatomy (Mechelli et al., 2004, Penicaud et al., 2012). Without further knowledge about the participants it is possible that these factors may have caused previous studies to overestimate the impact of deafness on the auditory cortex.

Finally, there is evidence that the FA is decreased, and MD and RD are increased in the deaf group in the motor thalamo-cortical tract. It is not clear why this would be the case, as the effects of congenital deafness on motor skills have not yet been investigated. Whilst all participants learnt sign language after the age of 10, the deaf group began to learn significantly earlier than the hearing. It is also possible that the groups differ in the extent of their usage, both of which may affect the motor thalamo-cortical tract. Allen et al. (2013) contrasted cortical volume in motor cortex in deaf signers, hearing signers and hearing control participants. They reported a trend towards leftward volume asymmetries in the deaf group, whereas in the hearing non-signing group the pattern was towards a rightward volume asymmetry in motor cortex, and in the hearing signing group a symmetrical pattern (Allen et al., 2013). They attribute this to activity dependent changes as a result of greater reliance on sign language in the deaf group (Allen et al., 2013). Finally, the motor thalamo-cortical tract includes contributions from axons involved in sensorimotor control of the mouth, which are necessary for speech production. Differences may exist between the deaf and hearing groups in speech usage. The deaf group do not integrate auditory feedback when they perceive speech. These reasons may contribute to the alterations observed in the motor thalamo-cortical tract.

There are several important caveats to bear in mind when interpreting DW-MRI data. Firstly, strong anatomical connections between regions do not necessarily correspond to equally important functional connections between regions (Johansen-Berg and Rushworth, 2009). We have endeavoured to link our results to findings from the behavioural and neuroimaging literature on deaf participants. There are many factors which can affect tractography results, including data quality, the distance between connected anatomical centres, as well as the complexity and geometry of the underlying fibres (Behrens et al., 2003, Behrens et al., 2007, Johansen-Berg and Rushworth, 2009, Jones et al., 2013). We addressed the issue of poor data quality through visual inspection of the data, which resulted in excluding three participants from further analysis. Poor quality data will tend to result in failure of paths to reach their cortical targets, rather than introducing any systematic error (Behrens et al., 2003). We thresholded data (60% of streamlines in each tract had to reach their cortical target) to try to reduce the impact of false positive connections between the seed region and cortical targets. Furthermore, the ‘winner takes all’ segmentation of cortical voxels into the cortico-thalamic tracts means that the contribution of voxels surrounding the thalamic area to microstructural measures is reduced. The limits of DW-MRI resolution mean that voxels in this region may contain genuine white matter connections to more than one cortical target, but the less strongly connected tracts are ignored for the purposes of extracting microstructural values. Whilst this may be considered a bias in data selection towards the more peripheral parts of the thalamo-cortical tracts, it ensures the independent sampling of tracts, necessary for investigating tract-specific group differences. Additionally, the physical proximity of the cortical target to the seed region will affect the ease with which a track is traced; tracts with a closer cortical target will necessarily have a greater probability associated with them. However, as we were contrasting tracts and thalamic parcellations derived from these between groups (rather than different tracts within the same brain), differences in tract connection probability related to cortical target proximity are unlikely to have systematically distorted results.

There are also caveats to be considered regarding the participants tested in the current study. Although animal models can be used to examine the influence of auditory deprivation, when considering humans, there is no perfect group contrast that allows the influence of auditory deprivation to be isolated from language experience. Previously, the majority of research into the effect of congenital deafness on brain anatomy or function in humans has contrasted deaf native signers with hearing native signers. This approach has the benefit of restricting aetiology of deafness to genetic causes and controlling for native exposure to a signed language. However, language experience inevitably differs between these groups as hearing native signers are more balanced sign/speech bilinguals than their deaf siblings. Furthermore, there is some evidence that hearing status interacts with native acquisition of sign language to influence the neural bases of visual motion processing (Bavelier et al., 2001, Neville and Lawson, 1987a). Sign language is a complex, dynamic visual stimulus, and it is possible that this form of ‘visual environmental enrichment’ will have a differential impact on deaf and hearing brains during early development.

We argue that a worthwhile contribution to this field is to contrast deaf and hearing individuals who have learnt a signed language later in life. However, this approach is also not without its drawbacks. Two of our deaf participants indicated they could not converse fluently with hearing people through speechreading alone. However, our findings were unchanged following analyses excluding these participants, demonstrating that our results were not due to insecure first language acquisition in the deaf group. Another drawback in research with individuals who are born deaf to hearing parents is the difficulty in controlling for aetiology of deafness, which is often unknown. A common cause of deafness in those with hearing parents is maternal rubella (Morzaria et al., 2004): five of the thirteen participants in the current study report this as the aetiology of their deafness. Intellectual disability caused by white matter lesions can also be a consequence of maternal rubella (Lane et al., 1996, Sugita et al., 1991). To reduce the chances of neurological problems or intellectual disability confounding our results, we sought deaf participants who were broadly matched in terms of education and occupational success to the hearing participants. In addition, all images were thoroughly screened for abnormalities. Whilst it is impossible to entirely rule out the possibility of undiagnosed neurological problems in this group, these steps minimize the risk that our group differences were driven by changes specific to those deaf through rubella. Concordance between results from studies which contrast deaf and hearing individuals with a range of different language backgrounds and different aetiologies will, in time, provide greater clarity regarding the true influence of auditory deprivation on brain anatomy and function.

Our findings demonstrate that congenital deafness causes plasticity in subcortical structures and thalamo-cortical projections, which ultimately have an effect on the control of information flow into and throughout the cortex. Microstructural measurements in the visual and frontal thalamic parcellations are altered in deafness, possibly suggesting more complex tissue in these regions, which may correspond to how visual information and visual attention is deployed differently by deaf people. Thalamo-cortical tracts to each cortical target, excluding temporal cortex, were altered. Differences in motor thalomo-cortical tracts may be linked to differences in speech, speech usage, age of sign language acquisition or sign language usage between the groups. Altered diffusivity of the somatosensory and occipital thalamo-cortical somatosensory tract may be the result of the enhanced somatosensory representation, and visual peripheral representation in deaf participants. Finally, changes to frontal and parietal connections may be the anatomical correlate of altered multi-modal perception and attentional control in the absence of sound. Thus the neural sequelae of congenital auditory deprivation can be observed throughout the brain and are not restricted to auditory cortex.
